# Molecular Markers of Influenza B Lineages and Clades

**DOI:** 10.3390/v6114437

**Published:** 2014-11-18

**Authors:** Rosaria Arvia, Fabiana Corcioli, Federica Pierucci, Alberta Azzi

**Affiliations:** Department of Experimental and Clinical Medicine, University of Florence, Viale Morgagni 48, 50134 Florence, Italy; E-Mails: rosaria.arvia@libero.it (R.A.); f.corcioli@virgilio.it (F.C.); fede_p85@alice.it (F.P.)

**Keywords:** influenza B viruses, molecular epidemiology, virus lineages and groups

## Abstract

Co-circulation of two influenza B virus lineages, B/Yamagata and B/Victoria, has been recognized since the late 1980s. The assessment of the prevalent lineage and the group of viruses in circulation is of importance in order to decide on the vaccine composition and evaluate its efficacy. The molecular characterization of influenza B viruses in circulation has been the aim of this study; this was approached by identifying and locating nucleotide substitutions in the influenza B virus hemagglutinin (HA) and neuraminidase (NA), specific for the lineage and/or clade. By the alignment of 3456 sequences from the influenza GISAID EpiFlu database, a high number of lineage- and group-specific nucleotide positions have been observed in the HA gene, but not in the NA gene. Additionally, an RT-PCR method has been developed, applicable directly to clinical specimens, which amplifies a short HA region that includes a group of unique molecular signatures. Twenty eight influenza B virus-positive respiratory specimens, collected in Tuscany in the seasons 2012–2013 and 2013–2014, were analyzed. The results revealed two clearly distinguishable patterns: one, more frequent, was characterized by all of the nucleotide changes associated with the B/Yamagata lineage (in most cases of Group 2), whereas the other exhibited all of the changes associated with the B/Victoria lineage. It can be concluded that the analysis of this short HA sequence can permit a rapid, highly sensitive determination of influenza B virus lineages and clades.

## 1. Introduction

Currently, influenza B viruses in circulation belong to two lineages distinct by their genetic and antigenic characteristics, which are referred to as the Yamagata and Victoria lineages, designated after their original isolates, B/Yamagata/16/88 and B/Victoria/2/87 [1,2]. These two lineages have co-circulated since the late 1980s [2]. Since 2008, most B/Victoria/2/87 lineage viruses have belonged to the B/Brisbane/60/2008 genetic clade (Group 1) [3] based on the hemagglutinin (HA) gene sequences. Instead, since 2007, the majority of B/Yamagata lineage viruses have been distributed into two main groups, with distinct genetic and antigenic characteristics, Group 2, represented by B/Brisbane/3/2007, and Group 3, represented by B/Bangladesh/3333/2007, which also contains the vaccine strain B/Wisconsin/01/2010 recommended for the season of 2012–2013 [4].

The assessment of the lineage and the group prevalent in circulation is of importance, in order to select the virus to be included in influenza vaccines and to evaluate the efficacy of vaccination. However, influenza B characterization is not commonly performed due to the underestimation of this information and to the lack of rapid and specific typing assays; the latter is performed by hemagglutination inhibition (HI) using suitable sera panels and/or by full-length sequencing of the HA gene.

As expected, by sequencing the HA and/or the NA genes of influenza B viruses, several amino acid substitutions, insertions and deletions can be detected [5,6,7,8,9,10]. HA protein sequences within each lineage show an identity higher than 97%, whereas inter-lineage sequence identity is on average 88%–90% [11,12]. However, there are few data in the literature reporting the detection of the lineage or group-specific molecular signatures [3,6].

The aim of this study was to identify and locate lineage- and/or clade-specific nucleotide substitutions in the HA and NA genes of influenza B viruses, to be used for the molecular characterization of the viruses in circulation. For this purpose, 3456 sequences of HA or NA genes of both the B/Victoria and B/Yamagata lineage viruses isolated between 2000 and 2013 have been aligned. A high number of lineage or clade characteristic nucleotide substitutions, located along the entire sequence of the HA gene, was identified. Our attention has been focused on a group of six nucleotide substitutions, located around an AAA insertion/deletion, occurring in a short sequence of about 60 nts.

## 2. Materials and Methods

### 2.1. Detection of Target Sequences in the HA and NA Gene for Influenza B Characterization

Two thousand nine hundred and fifty-six HA gene sequences and 500 NA gene sequences of influenza B viruses isolated between 2000 and 2013 have been downloaded from the influenza GISAID (Global Initiative in Sharing Avian Influenza Data) EpiFlu database, which reports also the lineage and, in many cases, the clade. Altogether, the 2956 HA sequences were 1460 from the B/Victoria and 1496 from the B/Yamagata lineage. These sequences were aligned using ClustalW v1.4 included in BioEdit v7.0.0.

### 2.2. Primer Design

The primers specific for the HA and NA target sequence of influenza B viruses were designed using Primer3, in order to amplify a short region encompassing the characteristic positions. The HA sequence primers were pF 5’GAC CCT ACA RAM TTG GAA CC’3 and pR 5’ART GGA ACC CCC AAA CRG TA’3, which amplify a 185-bp sequence, and for the NA gene, the forward and reverse primers were pF 5’GGG CAA AAT CCC AAC WGT Ag’3 and pR 5’GCA ATT GCA GGC ACT TTC TT’3, which amplify a 210-bp sequence.

### 2.3. Clinical Samples

During the influenza season of 2012–2013, a total of 376 respiratory samples were analyzed by RT real-time PCRs to detect influenza viruses A(H1N1) 2009, A(H3N2) and influenza virus type B [13]. Thirty-seven patients were positive for A(H1N1) 2009 pandemic virus; 3 were positive for A(H3N2); and 36 were positive for influenza B virus. In addition, during the last influenza season, a total of 311 respiratory samples were analyzed with the same objective. Twenty-nine patients were positive for A(H1N1) 2009; twenty-eight were positive for A(H3N2); and only 4 were positive for influenza B virus. The influenza type B isolates were further analyzed to verify if they could be clustered in discrete lineages and groups in circulation.

### 2.4. Viral RNAs Extraction and One-Step RT-PCR

Extraction of viral RNAs from clinical samples was carried out using a commercially available kit (E.Z.N.A. Viral RNA kit, Omega bio-tek, Norcross, GA, USA) according to the manufacturer’s instructions. Two one-step RT-PCRs have been developed, one for the amplification of target sequences on the HA gene and the other one for the amplification of target sequences on the NA gene. The QuantiTect virus kit, without ROX dye (Qiagen, Valencia, CA, USA), was used for each RT-PCR according to the manufacturer’s instructions.

After retro-transcription and denaturation, 40 cycles of amplification were performed (95 °C for 15 s, 55 °C for 30 s, 72 °C for 1 min), followed by a final extension at 72 °C for 7 min. The reaction volume was 25 µL (5 µL of 5X QuantiTect virus master mix, 0.25 µL of 100X QuantiTect virus RT mix, 0.5 µL of each primer [10 µM], 5 µL of extracted RNA and H_2_O to reach the final volume).

### 2.5. Sanger Sequencing and Phylogenetic Analysis

The PCR products were analyzed by dideoxy Sanger sequencing. Sequencing was carried on an ABI Prism 377 automatic sequencer (Applied Biosystems, Milan, Italy), using the ABI Prism Dye Terminator cycle sequencing Ready Reaction kit. Then the sequences obtained were aligned using ClustalW v1.4 included in BioEdit v7.0.0. A group of 18 influenza B viruses were cultured in MDCK cells, allowing us to perform the full sequencing of the HA1 gene. The phylogenetic analysis of these HA sequences was carried out using the neighbor joining (NJ) method (MEGA software, version 6.0). The parameter employed was p-distance, and the robustness of the internal branches was determined by 1000 bootstrap replications.

## 3. Results

**Figure 1:**
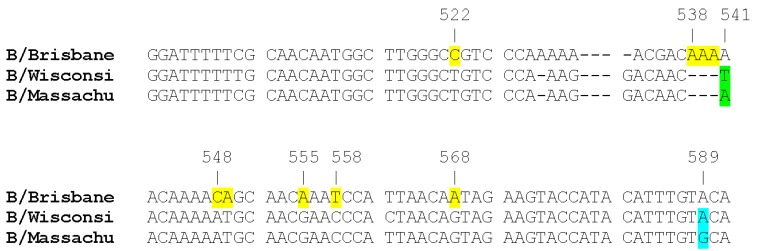
Nucleotide changes distinguishing the influenza B viruses of the B/Victoria lineage (here, represented by B/Brisbane/60/2008) from the viruses of the B/Yamagata lineage (here, represented by B/Wisconsin/1/2010, Group 3, and B/Massachusetts/2/2012, Group 2). The figure shows the changes present in a short region, about 60 nts long, of the HA, selected as described in the text. The lineage-specific markers (nts 522, 538–540, 548, 555, 558, 568) are highlighted in yellow, those that are clade specific (nt 541) in green. In blue is indicated another nucleotide substitution (nt 589), which characterizes the influenza B/Yamagata viruses of clade 2 from those of clade 3 analyzed in this study.

Two thousand nine hundred and fifty-six sequences of HA and 500 sequences of NA genes of influenza B viruses of both the B/Victoria and B/Yamagata lineage, isolated between 2000 and 2013, have been downloaded and aligned. About 100 lineage-specific molecular signatures were observed in the HA gene, and none in the NA gene. We focused our attention on a short region of the HA gene, encompassing seven lineage-specific molecular signatures, quite close to each other (Figure 1); in fact, using the numbering of B/Brisbane/60/2008, starting from the ATG codon, all of the viruses belonging to the B/Victoria lineage had an AAA insertion at the position 538–540 (corresponding to the amino acid position 180), which is deleted in the B/Yamagata lineage; moreover, B/Victoria lineage viruses at position 547–549 had the triplet ACA coding for threonine (T 183) and at position 558–560 the triplet TCC coding for serine (S 187) instead of AAT coding for asparagine (N) and of CCC coding for proline (P), respectively, which are characteristic of the B/Yamagata lineage viruses. Furthermore, most B/Victoria lineage viruses had a C at nucleotide position 522 (4% only had a T) and an A at nucleotide positions 555 and 568; instead the B/Yamagata lineage had, always, T, G and G in the same positions. As a group-specific signature, the triplet AAC at position 541–543 (coding for N at position 181) characterized the B/Yamagata lineage, Group 2, and the TAC (coding for tyrosine, Y) in the same position characterized the viruses of Group 3. As regards the NA gene, it was observed that at nucleotide position 600, in the triplet AGT, which codes for S at amino acid position 198, a G was associated mainly with the B/Yamagata lineage, whereas an A (AAT, which codes for N) was more frequently detected in the B/Victoria lineage. Near nucleotide position 600, at nucleotide position 596, a G to A modification creates the amino acid substitution D197N: this may be associated with reduced susceptibility to neuraminidase inhibitors (NAIs).

A total of 28 out of the 40 clinical samples (collected during the two influenza seasons) positive for type B influenza viruses could be amplified by the two one-step RT PCRs targeting short sequences of the HA and the NA gene. These reactions were unable to amplify specimens positive in the diagnostic RT real-time PCR (targeting the M gene) with a threshold cycle (Ct) > 37 (corresponding, approximately, to 12 copies/µL). The same sensitivity characterized, in our hands, the RT-PCR suggested by WHO (World Health Organization) [14], used as a reference method to establish the influenza B lineage. These 28 samples, with a Ct ranging between 20 and 37, were from both inpatients (11 adults and one child) and outpatients (14 children and two adults).

As Table 1 shows, in 8/28 (29%) samples, HA sequences with 538–540 ins AAA were found, as characteristic of the B/Victoria lineage; in the same eight HA sequences, the substitution N180K (lysine instead of asparagine) was present. This substitution is observed in the B/Brisbane/60/2008-like viruses, the group of the B/Victoria lineage in prevalent circulation since 2008. These eight viruses had also threonine in position 183 and serine in position 187, as well as a C at nucleotide position 522 and an A at nucleotide positions 555 and 568, confirming their inclusion in the B/Victoria lineage. The HA of the influenza B viruses found in the remaining 20 clinical samples (71%) (Table 1) was characterized by the 538–540 AAA deletion, which is a hallmark of the B/Yamagata lineage, as well as by the presence of the triplet AAT at position 547–549 (N 183) and CCC at position 558–560 (P 187); in addition, a T was present at nucleotide position 522 and G at nucleotide positions 555 and 568. In 17 out of these 20 viruses, the triplet AAC (N181), a hallmark of Group 2, was present at position 541–543. Three of these 20 had a T at position 541 (TAC coding for Y), characteristic of the B/Yamagata Group 3. By the analysis of the HA sequences downloaded from the GISAID EpiFlu database, at position 589, the presence of A was associated mainly with the B/Victoria lineage and with B/Yamagata clade 3; instead, the presence of G in the same position was associated with B/Yamagata clade 2. In the influenza B viruses analyzed in this study, 589 A was detected in all of the sequences with the pattern B/Victoria-like and only in three with the pattern B/Yamagata-like (characterized also by T 541), whereas in 17 at the same position, a G was present.

**Table 1:** Influenza B lineages and clades in Tuscany during the seasons 2012–2013 and 2013–2014.

Patient	HA Gene 522 538–540 541 548-549 555 558 568 589	NA Gene 600	Lineages (Clades)
1	C AAA A CA A T A A	A	Vic (1)
2	T Del A AT G C G G	G	Yam (2)
3	C AAA A CA A T A A	A	Vic (1)
4	T Del A AT G C G G	A	Yam (2)
5	T Del A AT G C G G	G	Yam (2)
6	T Del A AT G C G G	G	Yam (2)
7	T Del A AT G C G G	G	Yam (2)
8	T Del A AT G C G G	G	Yam (2)
9	T Del A AT G C G G	G	Yam (2)
10	T Del T AT G C G A	A	Yam (3)
11	C AAA A CA A T A A	A	Vic (1)
12	T Del A AT G C G G	G	Yam (2)
13	C AAA A CA A T A A	A	Vic (1)
14	C AAA A CA A T A A	A	Vic (1)
15	C AAA A CA A T A A	A	Vic (1)
16	T Del A AT G C G G	G	Yam (2)
17	T Del A AT G C G G	G	Yam (2)
18	T Del A AT G C G G	G	Yam (2)
19	C AAA A CA A T A A	A	Vic (1)
20	T Del A AT G C G G	G	Yam (2)
21	T Del A AT G C G G	G	Yam (2)
22	T Del A AT G C G G	G	Yam (2)
23	T Del A AT G C G G	G	Yam (2)
24	C AAA A CA A T A A	A	Vic (1)
25	T Del A AT G C G G	G	Yam (2)
26	T Del A AT G C G G	G	Yam (2)
27	T Del T AT G C G A	G	Yam (3)
28	T Del T AT G C G A	A	Yam (3)

**Figure 2:**
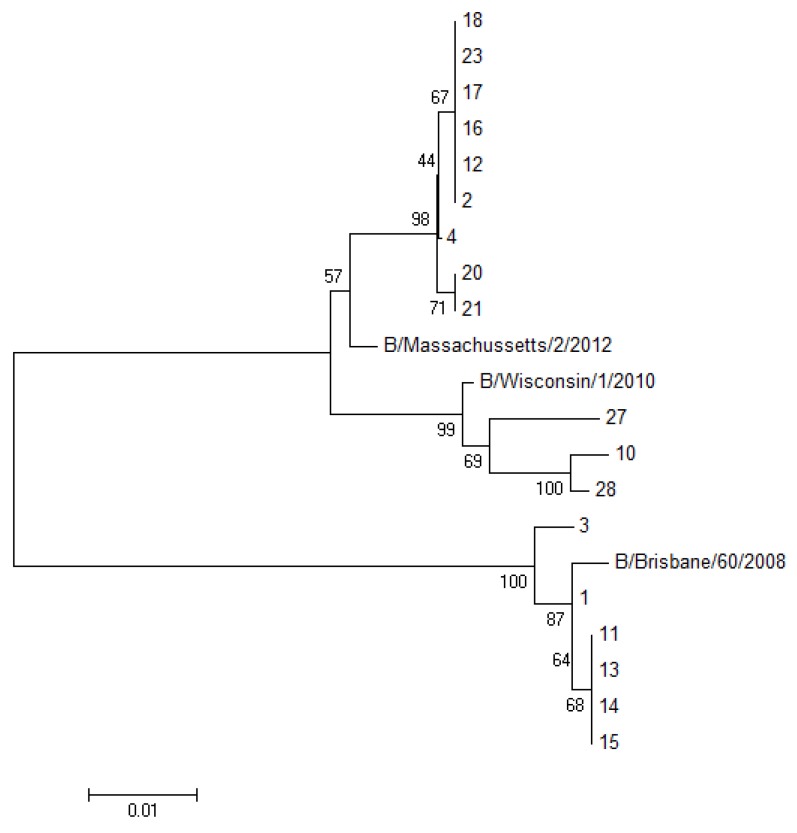
Phylogenetic tree of the HA1 portion from the HA gene of 18 sequenced samples analyzed with the neighbor joining algorithm. (The number of sequences in the tree corresponds to the patient’s number reported in Table 1).

NA sequencing of influenza B viruses from all clinical samples showed a G at nucleotide position 596, which codes for D (GAC) at amino acid position 197, suggesting that all of these viruses were sensitive to the NAIs. At nucleotide position 600, 17 clinical samples showed a G (AGT), which codes for S at amino acid position 198, more frequently associated with the B/Yamagata lineage, while eight clinical samples showed an A (AAT), which codes for N at amino acid position 198, more frequently associated with the B/Victoria lineage, in agreement with the results of the HA sequence analysis (Table 1). The sequence of NA gene from three other clinical samples showed N at amino acid position 198 (N198), whereas the sequences of the HA had a deletion at position 538–540 in the HA gene, as well as the other substitutions, characteristics of the B/Yamagata lineage. The complete sequencing of the HA and NA gene of two of these three viruses, performed at the National Institute for Medical Research (London, United Kingdom) and published in the GISAID EpiFlu database (Accession Numbers EPI464735, EPI464736 (B/Firenze/6/2013) and EPI530711, EPI530712 (B/Firenze/02/2014)), confirmed that they belong to the B/Yamagata lineage. From eighteen specimens, the virus could be isolated in MDCK cells. In these cases (16 related to the 2012–2013 influenza season and two to the 2013–2014 season), the results obtained with the method proposed here could be confirmed by Sanger sequencing of the full HA gene, using the primers suggested by WHO [14] and phylogenetic analysis (Figure 2).

## 4. Discussion

The differentiation between the two lineages of influenza B viruses is traditionally performed by HI. This assay is time-consuming, first requiring virus isolation and cultivation. Molecular assays aimed at detecting lineage- and/or clade-specific molecular markers could achieve the same result in less time, with high sensitivity. The comparison of 2,956 HA gene sequences from either the B/Victoria or the B/Yamagata lineage (clade 2 or clade 3) showed a high number of possible lineage- and/or clade-specific molecular markers, at variance with the NA gene sequences. The first difference observed was an AAA insertion/deletion at position 538–540 in the HA sequence able to distinguish between the two lineages. In a short sequence, 60 nts long, including this AAA ins/del, six other nucleotide substitutions, lineage- and/or clade-specific, were identified. The RT-PCR provides the amplification of the part of the HA gene, (185 nts long, encompassing the 60 nts-long target sequence), whose sequence can identify the lineage and clade of the influenza B viruses in circulation. This approach was used for the characterization of the influenza B viruses identified in Tuscany during the 2012–2013 and 2013–2014 influenza season. The 2012–2013 season was characterized by the frequent circulation of influenza A(H1N1) 2009 viruses and of type B viruses, while during the last influenza season, the circulation of influenza type B viruses in Tuscany seemed to be less frequent than that of influenza A viruses. The analysis of the short, amplified region of the HA gene showed two clearly distinguishable patterns. The more frequent pattern, observed in 20 cases, was characterized by the AAA deletion and by the presence of all of the substitutions associated with the B/Yamagata lineage; 17 on the basis of two further molecular signatures can be included in clade 2 and three in clade 3. The pattern of the remaining eight cases was characterized by the AAA insertion and by all of the substitutions associated with the B/Victoria lineage in this part of the gene. Thus, the analysis of the short HA sequence identified in this study allowed us to show that in the season of 2012–2013 in Tuscany, as in the other European countries, the two influenza B lineages co-circulated. The prevalent lineage was B/Yamagata, the lineage that included the strain B/Wisconsin/1/2010 recommended for the 2012–2013 trivalent vaccine. However, 94% of the B/Yamagata lineage viruses detected in this study, during the 2012–2013 influenza season, belonged to Group 2 and not to Group 3 (which includes the vaccine strain B/Wisconsin/1/2010). Anyway, the patient infected with this influenza B virus had not been vaccinated. The only two influenza B virus detected during the 2013–2014 influenza season belong to Group 3 and not to Group 2, which include the strain B/Massachusetts/2/2012 of the trivalent vaccine for this season.

During these epidemic seasons, 66 out of 639 patients were shown to be positive for influenza A(H1N1) 2009. Forty four were from inpatients (21 (47%) hospitalized in intensive care units, ICUs) and 22 from outpatients. Another 40 were positive for influenza B virus, 20 from inpatients, (two hospitalized in ICU (10%)) and 20 from outpatients. These data suggest that influenza B viruses may be frequently the cause of hospitalization. However, more severe forms, requiring hospitalization in ICUs, occur less frequently than following influenza A(H1N1) 2009 virus infection.

Forty six percent of influenza B virus infections, in this study, required hospitalization, independently of the lineage or clade. It is possible that the pathogenicity of type B influenza virus has been underestimated in the past. After the 2009 pandemic, patients hospitalized in different clinical settings are more frequently controlled for all types of influenza viruses using RT real-time PCR assays of high sensitivity, which could be the reason for the detection of a higher percentage of influenza B severe infections.

Different molecular methods have been developed for the characterization of influenza B viruses. These include mainly real-time PCR, which amplifies HA sequences using primers and probes specific for the two lineages [14,15,16], and pyrosequencing analysis of target regions in the HA and NA genes. Pyrosequencing analysis has been used also to detect simultaneously, in the NA gene, markers that distinguish between B/Yamagata and B/Victoria lineages and several mutations associated with NAIs [5]. In this study, two one-step RT-PCRs followed by sequencing were developed with the aim of detecting simultaneously molecular markers, both in the HA gene and NA gene, allowing one to distinguish and characterize the influenza B viruses. However, only the analysis of the HA gene sequence has given reliable results, in agreement with data in the literature [6]. In comparison with other published assays [5,6,14,15,16], the method described here has the advantage of simultaneously distinguishing the two main lineages of circulating influenza B viruses and the different groups within the lineages by a one-step RT-PCR targeting a small HA sequence. Only one method reported in the literature, based on pyrosequencing [6], is also able to distinguish the clades. However, it requires the use of a specific instrument not available in all laboratories.

The RT-PCR described here allowed also the analysis of clinical samples with a low viral load (12 copies/µL).

The importance of the characterization of influenza B viruses, and not only of influenza A viruses in circulation, is evident. In fact, on this basis, the WHO recommended for Northern Hemisphere influenza vaccine composition (for both seasons, 2013–2014 and 2014–2015) a change in the B/Yamagata lineage virus substituting the previous Group 3 vaccine component (B/Wisconsin/1/2010-like virus) with a Group 2 virus (B/Massachusetts/2/2012-like virus), due to increasing circulation of Group 2 B viruses [4]. Moreover, WHO has proposed to include in the quadrivalent vaccine an influenza B/Victoria virus related to the B/Brisbane/60/2008 virus.

As a consequence, there is the need for sensitive and suitable methods able to identify not only the lineage, but also the group of the type B influenza viruses in circulation, as with the assay described here, for diagnostic and epidemiological analyses.
